# Atezolizumab, a PD-L1 Inhibitor: An Association of Bleeding Gastric Ulcer With Its Use

**DOI:** 10.7759/cureus.15637

**Published:** 2021-06-14

**Authors:** Anusha Bapatla, Tooba Tariq, Maryam Bilal Haider, Bashar Mohamad

**Affiliations:** 1 Internal Medicine, Detroit Medical Center-Sinai Grace Hospital, Wayne State University, Detroit, USA; 2 Internal Medicine, California Institute of Behavioral Neurosciences & Psychology, Fairfield, USA; 3 Gastroenterology and Hepatology, Wayne State University, Detroit, USA; 4 Internal Medicine, Detroit Medical Center-Sinai Grace Hospital, Detroit, USA

**Keywords:** atezolizumab, immune check point inhibitors, upper gastrointestinal manifestations, gastrointestinal manifestations, gastric ulcer

## Abstract

Atezolizumab is a programmed death-ligand 1 inhibitor, an immune checkpoint inhibitor (ICI), useful in various advanced solid malignancies. As atezolizumab is more commonly used nowadays, physicians should be aware of the rare associated adverse events (AEs). Most of the AEs associated with the ICIs are immune-related, and the common gastrointestinal (GI) manifestations are colitis and diarrhea. Upper GI manifestations are rare with atezolizumab, and bleeding gastric ulcer is even rarer. We report here a case of a 62-year-old male with hepatocellular carcinoma who presented with upper GI bleed after atezolizumab therapy. Esophagogastroduodenoscopy showed multiple gastric ulcers, which are likely the cause of his bleeding.

## Introduction

Immune checkpoint inhibitors (ICIs) are in more common use recently for advanced solid malignancies due to high efficacy and low side-effect profile. Atezolizumab is a fully humanized monoclonal antibody to programmed death-ligand 1 (PD-L1). It selectively targets PD-L1 and prevents interaction with programmed cell death protein 1 (PD-1), thus reversing T cell suppression and increasing anti-tumor activity. Adverse events (AEs) associated with ICIs involve multiple organ systems, and the commonly postulated mechanism is immune dysregulation [1,2]. Well-reported gastrointestinal (GI) complications associated with atezolizumab and other ICIs are immune-mediated colitis, diarrhea, and autoimmune hepatitis [1,3,4]. Other GI complications associated are pancreatitis, celiac disease, constipation, nausea, and anorexia [1,5]. Bleeding gastric ulcers associated with ICIs including atezolizumab are uncommon, and only a handful of cases have been reported. Here, we present the case of a 62-year-old male who was diagnosed with a history of hepatocellular carcinoma (HCC) (on atezolizumab) who presented with melena. Esophagogastroduodenoscopy (EGD) showed multiple gastric ulcers with stigmata of recent bleeding.

## Case presentation

**Figure 1:**
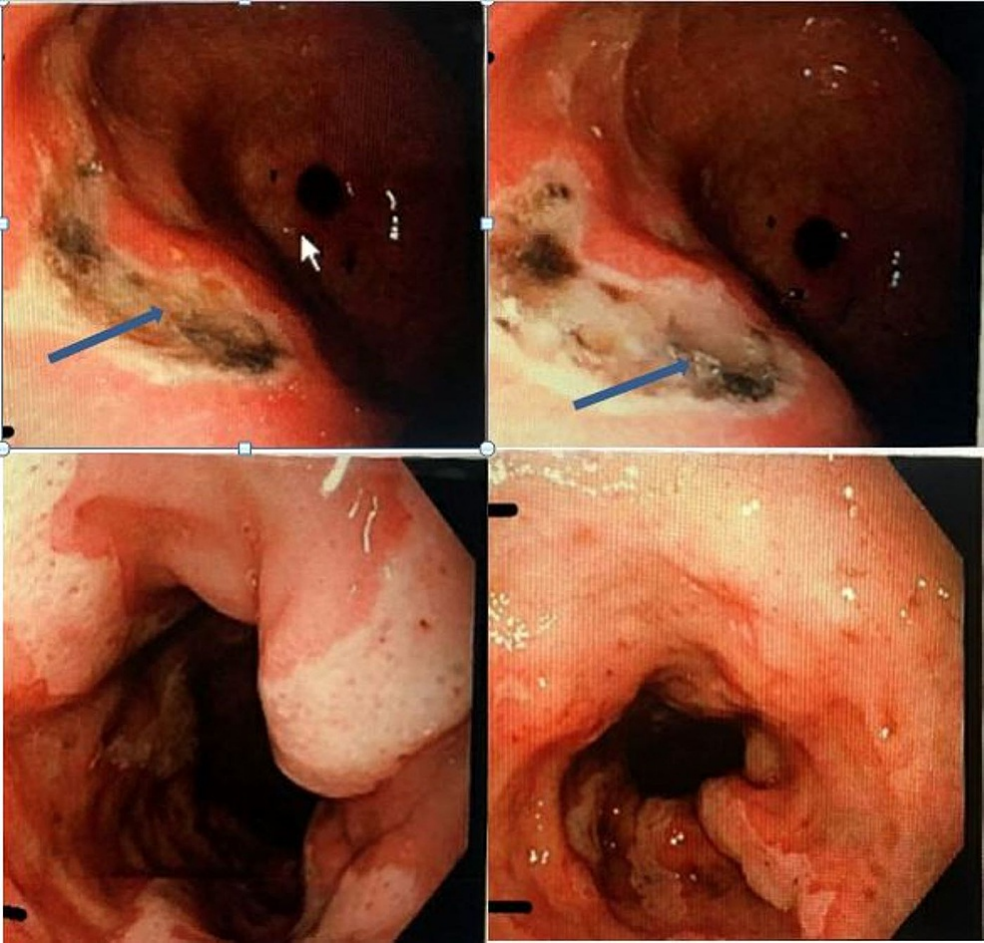
Esophagogastroduodenoscopy showing multiple gastric ulcers with hematin spots (Forrest IIC classification) noted

A 62-year-old male with a history of hepatitis C cirrhosis complicated by HCC and portal vein thrombosis on rivaroxaban presented to the hospital with melena for three days. The patient endorsed some mild generalized cramping abdominal pain, and denied any dizziness, syncope, nausea, vomiting, or hematemesis. The patient had a history of esophageal varices that were banded six years ago. He was diagnosed with HCC a year ago and was first started on levatinib; however, it was stopped since the patient developed neuropathy. The patient was initiated on atezolizumab and had received two doses with the last dose given three weeks before presentation. On presentation, the patient was tachycardic in 110s, hemoglobin was 8.4 g/dL (from baseline of 14-15), international normalized ratio 1.32, prothrombin time 13.9, and platelet count was 79,000. CT of the abdomen and pelvis showed a stable left hepatic mass. EGD showed four columns of large non-bleeding esophageal varices that were banded. Multiple gastric antral ulcers and prepyloric ulcer with dark spots were noted, with the largest ulcer measuring up to 2 cm (Figure 1). Multiple clean-based duodenal ulcers were also noted.

He was not on non-steroidal anti-inflammatory drugs (NSAIDS), steroids, or any other drugs that can cause gastric ulcer. The patient’s GI bleed was attributed to the gastric ulcers, which were thought to be secondary to the immunotherapy agent, atezolizumab. The patient was continued on proton pump inhibitors (PPIs), and octreotide drips for a total of 72 hours and later switched to PPI 40 mg BID. Rivaroxaban was held for a week due to the findings of extensive gastric ulceration on EGD. The patient was seen in the oncology clinic a week later with no recurrence of bleeding, and his anticoagulation was restarted. Patient was restarted on atezolizumab three weeks later and continued on PPI twice a day without any evidence of further GI bleed.

## Discussion

ICIs are monoclonal antibodies that target immune checkpoints, such as cytotoxic T-lymphocyte-associated protein 4, PD-1, or PD-L1. Atezolizumab is a humanized monoclonal antibody against PD-L1 indicated for advanced solid organ malignancies. AEs associated with ICIs are mainly due to immune dysregulation [1,2]. These immune-related adverse effects can affect any organ, and the most commonly involved systems are dermatology, endocrinology, and luminal GI tract [2,6]. Less commonly affected organ systems are cardiovascular, pulmonary, renal, and musculoskeletal [2,6].

Well-known and well-studied luminal GI AEs are colitis and diarrhea [1,3,4]. Upper GI AEs are less common, and most commonly reported among them are nausea and decreased appetite [1,5]. Other less commonly reported upper GI complications include mucositis, gastritis, and esophagitis [5]. Bleeding gastric ulcer associated with atezolizumab is very rarely reported. Tang et al. in their study of 60 patients with endoscopy and biopsy showed a correlation between ICIs and upper GI inflammation after ruling out other risk factors [7]. Fazal et al. in their study of 25 patients showed common upper GI features, which include erythema, edema, and friability [8]. Irshaid et al. demonstrated that upper GI inflammation is similar to *Helicobacter pylori* gastritis and celiac disease with minor variations on histology [9]. Above studies support immune dysregulation and activation of T cell lymphocytes as a possible mechanism for the development of upper GI ulcer with ICIs [7-9].

In this case, atezolizumab was given for advanced HCC after discontinuing levatinib due to associated neuropathy. First-line systemic therapy options available for advanced HCC include sorafenib, levatinib, or atezolizumab combined with bevacizumab [10]. Our patient had melena with hemodynamic instability and acute drop in hemoglobin, likely due to bleeding from gastric ulcers. Other risk factors such as NSAID and steroid use were absent; hence, atezolizumab was deemed the likely culprit given the temporal relationship between initiation of immunotherapy and onset of symptoms. 

Swei et al. in their case report of a 71-year-old male with metastatic urothelial carcinoma who had odynophagia after two doses of atezolizumab showed LA grade 4 esophagitis, diffuse gastritis, and severe duodenitis on EGD. These findings were attributed to atezolizumab after other etiologies were ruled out [11]. Fujji et al. reported a case of a middle-aged man who developed cholangitis and gastritis after atezolizumab. EGD showed erosive gastritis and erythema and they were confirmed with a biopsy [12]. Similar to the above cases, our case also demonstrated gastric and duodenal inflammation and ulcer; however, our case presented with GI bleed in the setting of anticoagulant use.

As per current guidelines, treatment of AEs associated with ICIs depends on the grade of AEs. Low-grade AEs can be treated with conservative management without any steroids or any discontinuation of ICIs. High-grade AEs may require discontinuation of immunotherapy and oral or intravenous steroids [5,13,14]. The use of glucocorticoids in ICI-induced gastric ulcer is controversial as steroids increase gastric ulcers and subsequent hemorrhage [5]. Tang et al. in their study of 60 patients showed that most of the patients with isolated GI manifestations respond well with PPIs or H2 receptor blockers [7]. Our patient responded well with conservative management, which includes PPIs and octreotide without any steroids. Resumption of immunotherapy depends on many factors, which include the tumor status, functional status of the patient, severity of AEs, and response of AEs to the treatment, and, hence, treatment resumption should be individualized [3]. Use of PPIs for the prophylactic use while initiating atezolizumab or while rechallenging atezolizumab is not well explored. Our patient remained symptom-free with high-dose PPIs and restarted atezolizumab without any further complications.

## Conclusions

To conclude, physicians should consider upper GI ulcer as a potential complication with atezolizumab and associated upper GI bleed, especially when the patient is on anticoagulation. Further research on the pathophysiology of upper GI ulcers associated with atezolizumab is needed to formulate management strategies better. We also recommend further studies on the prophylactic use of PPIs with initiation of atezolizumab and while rechallenging with atezolizumab.
